# Towards Robust UAV Navigation in Agriculture: Key Technologies, Application, and Future Directions

**DOI:** 10.3390/plants15091303

**Published:** 2026-04-23

**Authors:** Guantong Dong, Xiuhua Lou, Haihua Wang

**Affiliations:** 1 College of Engineering, China Agricultural University, Qinghua East Road No. 17, Haidian, Beijing 100083, China; 2023307140125@cau.edu.cn; 2 College of Information and Electrical Engineering, China Agricultural University, 17 Qinghua East Road, Haidian, Beijing 100083, China

**Keywords:** UAV, UAV navigation, precision agriculture, localization and mapping, path planning, precision spraying

## Abstract

Unmanned aerial vehicles (UAVs) are becoming an important platform for precision agriculture, supporting both high-throughput sensing and active field operations such as spraying, monitoring, and phenotyping. However, unlike general UAV applications, agricultural environments impose distinctive challenges due to heterogeneous field structures, canopy occlusion, terrain variation, dynamic disturbances, and strong coupling between navigation performance and task quality. To address this gap, this review presents a systematic analysis of UAV navigation in agricultural environments from a system-level perspective. The review first summarizes the core technical components of agricultural UAV navigation, including sensing, localization, mapping, planning, and control. It then discusses how navigation requirements vary across representative scenarios such as open fields, orchards, and terraced farmland, and examines their roles in key applications including aerial mapping, field monitoring, precision spraying, and close-range orchard operations. In addition, datasets, simulation platforms, and evaluation protocols relevant to agricultural UAV navigation are reviewed. Finally, major challenges are identified, including scene heterogeneity, perception degradation, insufficient task-semantic integration, limited control robustness, and the lack of standardized benchmarks. Future research should move toward robust, task-aware, and modular navigation architectures that support reliable and scalable agricultural UAV deployment.

## 1. Introduction

With continued advances in productivity and agricultural technology, global population growth is placing increasing pressure on food security [1]. The world population is projected to exceed 9 billion by 2050 and may surpass 10 billion by the end of this century [1,2,3]. Under such pressure, agricultural systems must improve productivity under limited land, water, and labor resources, making agricultural automation and precision agriculture increasingly important for enhancing resource-use efficiency and production resilience [4,5,6]. Precision agriculture relies on continuous sensing, quantitative analysis, and data-driven decision-making to support refined crop and field management [7,8,9,10,11]. In parallel, rapid progress in automation, computer vision, and artificial intelligence [12,13,14,15,16,17,18,19,20,21,22,23,24,25,26,27] has accelerated the deployment of unmanned aerial vehicles (UAVs) as flexible platforms for aerial sensing and field operations across the agricultural production chain [28,29].

Because of their high maneuverability, low-altitude accessibility, and relatively low deployment cost, UAVs have been widely adopted not only in agriculture but also in forestry, geospatial applications, and urban remote sensing [30,31,32,33,34,35]. In these domains, UAVs support tasks such as object detection, mapping, search, and 3D modeling [30,34,36,37,38]. More broadly, practical UAV systems typically rely on onboard vision and related sensors to perceive the environment, support autonomous navigation, and enable task execution [28,39].

In recent years, UAV applications in agriculture have expanded from remote sensing tools to active operational platforms involved in agricultural production. In monitoring tasks, UAVs have been widely used for crop growth assessment, abiotic stress identification, biotic stress detection, and yield estimation, thereby supporting high-throughput phenotyping, breeding, and agricultural decision-making [40,41,42,43,44,45]. Beyond monitoring, UAVs have also progressed in active operations such as precision spraying. Representative studies have demonstrated UAV-based closed-loop workflows that integrate sensing, prescription generation, and spray control, highlighting the transition of UAVs from passive observation platforms to operational systems for agricultural intervention [46,47].

Despite this progress, agricultural UAV deployment remains less mature than UAV applications in non-agricultural domains such as urban mapping, inspection, and general remote sensing, because agricultural environments impose distinct task requirements, engineering constraints, and environmental disturbances [48,49,50]. Unlike many general UAV applications that focus primarily on observation or data acquisition, agricultural UAVs often require tight coupling between sensing, decision-making, and physical task execution. This is particularly evident in operations such as spraying, where payload variation, liquid sloshing, and wind disturbances increase dynamic uncertainty and control difficulty [51]. In addition, agricultural task quality is highly sensitive to flight altitude, speed, and path deviation, since these factors directly affect deposition uniformity, coverage quality, and drift risk [52]. Large-area operations under limited endurance further make coverage planning a key challenge [53], while near-ground flight in the presence of irregular field boundaries, vegetation occlusion, airflow, and dust places higher demands on perception robustness and flight stability [54].

Against this background, navigation is becoming a foundational capability for agricultural UAVs to move from being merely flyable and observable to being reliably task-executable. High-precision localization and trajectory tracking directly affect operational repeatability and spraying consistency, while autonomous agricultural operation further requires the integration of perception, planning, and control under complex environmental conditions [28,46,55,56]. More broadly, agricultural UAV systems are increasingly evolving toward a closed-loop precision-operation paradigm that links perception, decision-making, and execution [57].

Existing reviews have examined agricultural UAVs from multiple perspectives, but most remain focused on application-level themes such as remote sensing, crop monitoring, deep learning-based perception, or spraying systems [44,45,54]. Even broader reviews of agricultural UAVs often treat navigation as an auxiliary module rather than as a system-level problem involving the coordinated interaction of perception, localization, mapping, planning, control, and safety [48]. By contrast, the non-agricultural UAV literature contains a much richer body of work specifically dedicated to autonomous navigation [28]. Therefore, a systematic review of UAV navigation in agricultural environments from a system perspective is still lacking.

To address this gap, this paper presents a systematic review of UAV navigation in agricultural environments from a system perspective (Appendix A). The specific objectives are as follows:

1. to systematically review and classify the key technological components of UAV navigation in agricultural environments;
2. to analyze the applicability and development trends of different navigation technologies in relation to representative agricultural operational requirements;
3. to summarize the major challenges in current agricultural UAV navigation research and discuss possible future directions.

The remainder of this paper is organized as follows. Section 2 describes the key technologies for UAV navigation in agricultural environments. Section 3 discusses the applications of UAV navigation in agricultural environments. Section 4 reviews datasets, simulation platforms, and evaluation protocols for agricultural UAV navigation. Section 5 highlights key challenges and future perspectives. Section 6 concludes the paper.

**Figure 1:**
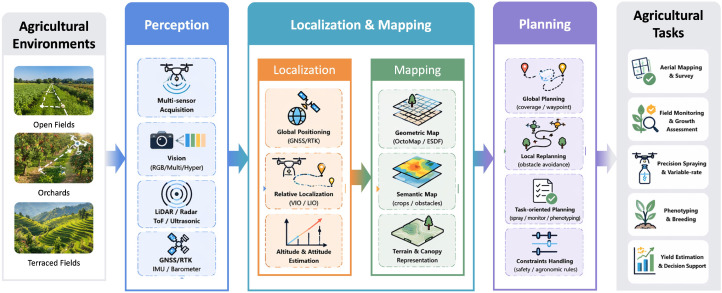
Overview of the hierarchical framework of agricultural UAV navigation and its representative task applications.

To provide a system-level overview of the scope of this review, Figure 1 summarizes the hierarchical organization of agricultural UAV navigation, including the relationships among agricultural environments, perception, localization and mapping, planning, and representative task applications. This framework serves as a conceptual roadmap for the subsequent sections.

## 2. Key Technologies for UAV Navigation in Agricultural Environments

### 2.1. Agricultural Operational Scenarios and Navigation Requirements

**Figure 2:**
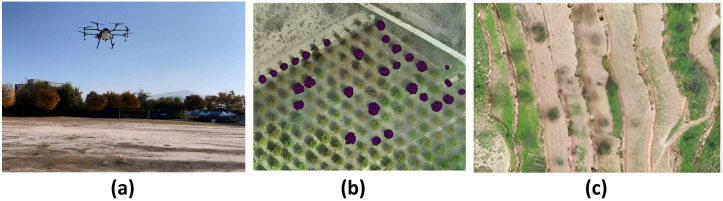
UAV operation scenarios. (**a**) Open fields [46]; (**b**) Orchard [58]; (**c**) Terraced fields [59].

The navigation requirements of agricultural UAVs are largely determined by operational scenarios. Different agricultural environments vary substantially in spatial structure, operational mode, and environmental disturbances, leading to distinct technical demands on localization accuracy, path planning, and flight control. In particular, environmental factors such as wind shear or airflow disturbances, GNSS signal degradation, and dense canopy occlusion can directly affect state estimation, perception reliability, and trajectory stability, and therefore should be regarded as core determinants of navigation performance in agricultural UAV systems. Figure 2 illustrates three representative UAV operational scenarios in agriculture: open fields, orchards, and terraced or mountainous farmlands. These scenarios not only cover the main application types of agricultural UAVs, but also reflect the typical technical characteristics of navigation problems in agricultural environments.

In typical agricultural operations, different environments impose differentiated requirements on UAV navigation systems (Figure 2). In open-field environments (Figure 2a), UAVs are commonly used for large-area crop protection spraying, variable-rate application, and field monitoring [46]. Because spraying is inherently position-sensitive, deviations in flight trajectory as well as fluctuations in altitude or speed can directly affect droplet coverage uniformity and deposition performance. Therefore, high-precision localization and stable trajectory tracking are essential for closed-loop spraying control. Meanwhile, large-scale continuous operations place higher demands on coverage path planning and trajectory stability, while wind disturbances, including low-altitude gusts and wind-shear effects over large open areas, together with payload variations, further increase navigation uncertainty and trajectory-tracking difficulty [46]. In orchards and forestry environments (Figure 2b), UAVs are mainly used for high-resolution data acquisition and plant-level management [58]. Such applications typically require repeatable flight paths and stable positioning to enable multi-temporal data registration. However, dense canopy occlusion, repetitive textures, and partial GNSS degradation beneath or near tree canopies often degrade both visual and satellite-based localization performance, thereby imposing greater demands on 3D environmental perception and robust localization [58]. In terraced or mountainous farmlands (Figure 2c), complex terrain makes conventional surveying difficult to implement efficiently, whereas UAV photogrammetry can provide high-resolution topographic information [59]. However, pronounced terrain variation and fragmented field structures require navigation systems to maintain stable localization and terrain-following flight capability in order to ensure flight safety and data quality. GNSS blockage and airflow disturbances may further reduce navigation stability [59].

Overall, agricultural UAVs are evolving from information acquisition tools into operational platforms for agricultural production, and navigation systems form the key foundation for stable operation. Different scenarios emphasize different navigation capabilities: open fields prioritize trajectory stability and coverage efficiency, orchards emphasize 3D perception and repeatable flight paths, and terraced fields require terrain adaptability and flight safety. Accordingly, agricultural UAV navigation typically relies on multi-sensor fusion and multi-layer navigation strategies to ensure robust operation. Based on these scenario-specific requirements, the next section discusses the key sensing technologies for agricultural UAV navigation. Although this review mainly focuses on representative open agricultural scenarios, confined agricultural environments such as greenhouses and plant factories, where GNSS is denied and navigation must place greater emphasis on alternative positioning and disturbance-rejection control, also represent an important direction for future agricultural UAV research [60].

### 2.2. UAV Sensors

**Figure 3:**
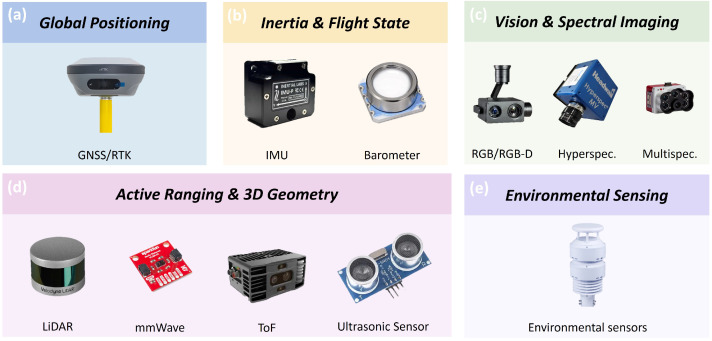
Representative sensors relevant to agricultural UAV navigation: (**a**) for global positioning; (**b**) for inertia & flight state; (**c**) for visual & spectral imaging; (**d**) for active ranging & 3D geometry; (**e**) for environmental sensing.

Autonomous navigation of agricultural UAVs depends on multi-sensor fusion for localization, environmental perception, and state estimation [61]. Considering both agricultural remote sensing payloads and operational characteristics, sensors relevant to agricultural UAV navigation can be grouped into five categories (Figure 3): (i) global positioning sensors, such as GNSS and RTK (Real-Time Kinematic); (ii) inertial and flight-state sensors, such as the Inertial Measurement Unit (IMU) and barometer; (iii) visual and spectral imaging sensors, such as RGB, multispectral, and hyperspectral cameras; (iv) active ranging and 3D perception sensors, including LiDAR (Light Detection and Ranging), millimeter-wave radar, ToF, and ultrasonic sensors; and (v) environmental disturbance sensing sensors, such as anemometers for wind speed and direction.

GNSS/RTK provides absolute position references in a global coordinate frame and serves as the fundamental localization method for agricultural UAV navigation systems. However, its accuracy can degrade under vegetation occlusion or complex terrain, so it is commonly fused with other sensors to improve robustness [61]. IMUs and barometers are used for flight-state estimation and closed-loop control input. The IMU provides high-frequency motion information but suffers from drift, and thus typically requires correction from external localization sources [61,62]. Visual and spectral imaging sensors are among the most common onboard devices for agricultural UAVs. RGB cameras support visual odometry and environmental perception and can provide complementary localization information in GNSS-limited environments [63]. Multispectral and hyperspectral sensors are primarily used for agricultural monitoring and prescription generation, although flight path repeatability and attitude stability directly affect data consistency [48,64,65]. Active ranging sensors support obstacle avoidance, altitude maintenance, and mapping by providing distance or geometric information, including LiDAR, millimeter-wave radar, ToF, and ultrasonic sensors [61]. Among these, LiDAR is suitable for high-precision 3D mapping; millimeter-wave radar is more robust under low-light or dusty conditions [66]; and ToF and ultrasonic sensors are commonly used for short-range distance measurement and altitude control [67]. In addition, ultrasonic anemometers are often used for onboard wind measurement to support the stability assessment of spraying operations.

Compared with general-purpose UAVs, agricultural UAVs often carry spraying systems or remote sensing payloads, and their variable payloads can alter system dynamics and increase uncertainty in navigation and control. As a result, stable agricultural UAV operation generally depends on multi-sensor fusion. The next section further discusses localization and state estimation methods for agricultural UAV navigation.

### 2.3. Localization

In autonomous UAV navigation systems, localization is the core state-estimation component linking perception, planning, and control in a closed loop [68]. Its objective is to provide a reliable absolute pose reference for global path planning before takeoff and to continuously estimate the UAV’s 6-DoF pose during flight [69], together with its relative relationship to maps or environmental elements such as crop rows, terrain, and obstacles, thereby supporting online replanning, trajectory tracking, and obstacle avoidance [70]. Given the environmental characteristics and operational requirements of agricultural scenarios, current mainstream localization methods can be broadly categorized into three groups: (i) GNSS-RTK-based absolute localization; (ii) relative localization based on vision, inertia, and LiDAR (e.g., VIO, SLAM, and LIO); and (iii) altitude estimation and terrain-following methods for operational safety.

**Figure 4:**
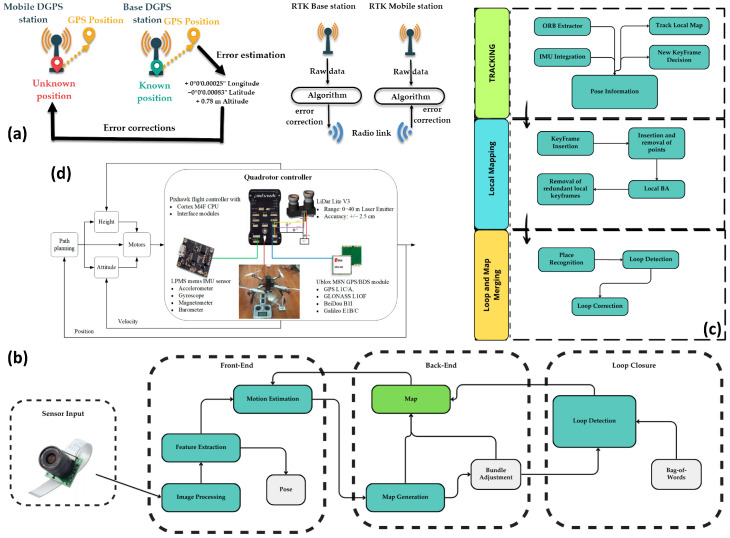
Representative localization frameworks for agricultural UAV navigation. (**a**) Principle of GNSS-RTK and differential positioning [72]; (**b**) General workflow of Visual SLAM [81]; (**c**) ORB-SLAM architecture with loop closure and multi-map support [81]; (**d**) Multi-sensor fusion architecture for position, attitude, and terrain-following height control [82].

In open-field agricultural scenarios, GNSS-RTK is one of the most widely used localization approaches (Figure 4a), as it can directly provide centimeter-level absolute coordinates [71] and is well suited to route tracking, field-boundary constraints, and precision spraying control [72]. Common implementations include onboard RTK and network RTK (VRS), often fused with IMU and compass data through extended Kalman filtering (EKF) or factor-graph methods [72,73]. This fusion combines high-frequency attitude information with low-frequency absolute positioning, thereby improving control stability and georeferencing consistency [74,75]. It also reduces the risks associated with IMU drift and single-sensor failure [76]. In agricultural surveying and precision mapping, RTK supports direct georeferencing, reduces dependence on ground control points (GCPs), and improves mapping efficiency [77]. Network RTK has been shown to provide uniform centimeter-level accuracy over distances of tens of kilometers [78,79]. However, GNSS remains susceptible to occlusion, multipath effects, and ionospheric interference [80], and its performance may deteriorate in orchards, greenhouses, or hilly environments, making integration with relative localization methods necessary.

When GNSS is unreliable or when higher-frequency and smoother pose estimates are required, visual-inertial odometry (VIO) [83] (Figure 4b) and visual SLAM [81] (Figure 4c) become core navigation approaches. Optimization-based methods, such as OKVIS [84], tightly couple visual and IMU constraints through sliding-window nonlinear optimization [69], enabling continuous real-time 6-DoF pose estimation [81,85]. Filtering-based methods, such as MSCKF [86] and Real-time Visual Odometry and Mapping [87], use EKF to achieve high-frequency state estimation with relatively low computational cost. In addition, loop closure and pose graph optimization, as in ORB-SLAM3 [88], can reduce long-range drift and improve global consistency. However, in agricultural environments, repetitive textures, illumination variation, and dynamic vegetation often degrade pure vision-based methods, making drift accumulation and localization instability more pronounced during long-duration operation. To address this limitation, LiDAR–Inertial Odometry (LIO) approaches such as FAST-LIO use tightly coupled iterative EKF fusion of LiDAR and IMU measurements to improve robustness in weak-texture and vegetation-disturbed environments [89]. Compared with VIO and visual SLAM, LIO is generally more robust under degraded agricultural conditions, but it usually imposes higher requirements on sensing hardware, payload, and system complexity. Agricultural multi-sensor datasets such as AgriLiRa4D further support the applicability of multimodal SLAM for orchard inspection and inter-row navigation [90].

In practical agricultural UAV deployment, however, localization methods must also satisfy strict onboard constraints in computation, memory, payload, and energy consumption [91]. For this reason, lightweight SLAM and state-estimation frameworks are becoming increasingly important for engineering implementation [91,92]. Compared with high-complexity mapping and optimization pipelines, deployable agricultural UAV systems often require more compact front-end processing, efficient sensor selection and fusion, and reduced map or feature representations, so as to maintain real-time pose estimation under limited onboard resources. Therefore, future agricultural UAV localization research should not only pursue accuracy and robustness in degraded environments, but also explicitly consider computational efficiency, memory footprint, and power-aware deployment for long-duration field operation [93].

In agricultural operations, altitude control is often more critical than planar position. Maintaining a constant distance from the canopy directly affects spraying uniformity, while aerial surveying requires stable flight altitude. Common approaches fuse barometer readings, GNSS altitude, and downward-looking ranging sensors such as ultrasonic, infrared, or laser sensors (Figure 4d) [82]. Barometers provide smooth short-term estimates but are prone to drift, GNSS altitude is affected by multipath interference [62], and downward-looking range sensors are most accurate at low altitude but are constrained by measurement range and surface reflectance. Accordingly, EKF or complementary filtering is typically used to fuse multiple altitude sources [94]. In environments with pronounced terrain variation or unreliable GNSS, terrain matching methods such as TERCOM/TRN can be used to improve navigation robustness by matching onboard altitude profiles with digital elevation models [95]. Such methods are particularly suitable for route following in hilly terraced fields, constant-height spraying in orchards, and inspection tasks over undulating terrain [82]. Table 1 summarizes localization methods for agricultural UAV navigation.

### 2.4. Mapping and Representation

In autonomous UAV navigation systems, the core objective of mapping is to transform sensor observations into environmental representations that can support path planning and obstacle avoidance [96,97,98]. Compared with conventional robotic environments, agricultural scenes are characterized by large scale, repetitive structures, and dynamic vegetation. Therefore, map representations must not only describe environmental geometry, but also support real-time updating and task-level decision-making. Existing studies mainly focus on three types of map representation: geometric maps, visual reconstruction maps, and semantic and dynamic maps.

**Figure 5:**
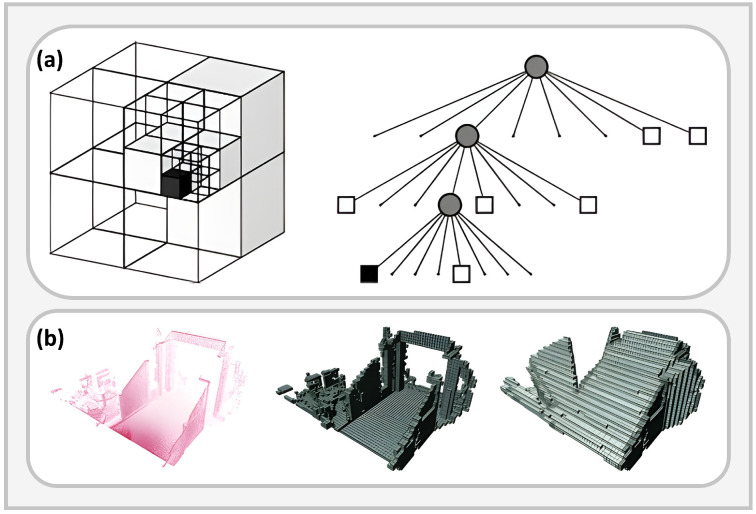
OctoMap representation of 3D occupancy grids based on octree structures. (**a**) Principle of octree storage for occupied and free cells. (**b**) Example octree map generated from point-cloud observations [102].

Geometric maps constitute the most basic form of environmental representation for UAV navigation, with occupancy grids, voxel maps, and their 3D extensions being the most common [99,100,101]. A representative example is OctoMap, which recursively represents spatial occupancy probabilities using an octree structure and can efficiently distinguish occupied, free, and unknown space while supporting efficient storage and querying in large-scale 3D environments (Figure 5) [102]. In agricultural scenarios, such maps are commonly used for low-altitude obstacle avoidance, farm-road identification, and spatial representation of field obstacles.

**Figure 6:**
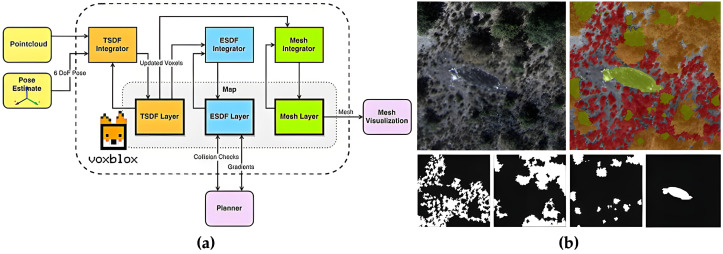
Representative mapping representations for agricultural UAV navigation: (**a**) structural diagram of the Voxblox system [105]; (**b**) example of a semantic map with labeled regions and corresponding binary masks [107].

However, occupancy maps provide only reachability information and are therefore insufficient for direct trajectory optimization. Many systems further construct Euclidean Signed Distance Fields (ESDFs), which encode obstacle distance and gradient information for planning [103]. Representative methods such as Voxblox incrementally generate ESDFs from truncated signed distance fields to support real-time distance queries, while FIESTA improves update efficiency for online replanning through a BFS-based incremental strategy [104,105,106]. As illustrated in Figure 6a, such distance-field representations are particularly suitable for near-ground low-altitude flight and canopy-proximal obstacle avoidance in agricultural UAV applications.

Another important class of maps is derived from visual SLAM, which can be broadly divided into sparse feature maps and dense or semi-dense depth maps [108]. Sparse maps, represented by ORB-SLAM2 [109], maintain keyframes and a limited number of 3D feature points, offering low computational cost and supporting real-time localization, but providing limited geometric completeness for fine obstacle avoidance or close-range operation. By contrast, dense or semi-dense maps provide more continuous geometric structure and are therefore more suitable for near-ground low-altitude applications. Typical examples include RGB-D reconstruction methods such as KinectFusion [110], multi-view stereo (MVS) reconstruction [111], and semi-dense methods such as LSD-SLAM [112]. These representations can better describe canopy geometry and obstacle distance, which is particularly valuable for orchard inspection and canopy-proximal flight.

As agricultural UAV navigation evolves from passability-oriented navigation to task-driven navigation, geometry alone is no longer sufficient for decision-making, and semantic mapping is becoming increasingly important. Metric-semantic mapping frameworks combine geometric reconstruction with semantic labels to produce maps that explicitly encode task-relevant elements such as crop rows, canopies, roads, ditches, utility poles, and other risk-related objects [107,113]. As shown in Figure 6b, such semantic representations provide stronger support for task planning and safety-aware navigation in agricultural environments.

In LiDAR-based mapping systems, point-cloud preprocessing is also critical to map quality. Common steps include ground segmentation [114], outlier removal [115], and voxel downsampling [116], which improve the stability of occupancy-map or ESDF updates [104]. For agricultural environments, dedicated farmland point-cloud filtering methods have also been developed. For example, Liu et al. [117] combined elevation-frequency histograms with a multi-feature Gaussian Mixture Model (GMM) to separate ground points, and further integrated geometric, intensity, and spectral features to improve ground-segmentation accuracy, thereby enhancing map construction quality in complex farmland environments. From an engineering perspective, mapping representations differ not only in geometric expressiveness and planning utility, but also in their suitability for resource-constrained onboard deployment. In agricultural UAV systems, this distinction is particularly important because onboard computing power, memory, payload, and energy are often limited. As summarized in Table 2, occupancy and sparse visual maps are generally more compatible with lightweight onboard deployment, whereas ESDF-based, dense, semantic, and dynamic representations typically impose higher memory, update, and inference costs. Therefore, the choice of mapping representation should be jointly determined by task requirements, scene complexity, and real-time processing constraints.

### 2.5. Path Planning and Obstacle Avoidance

**Figure 7:**
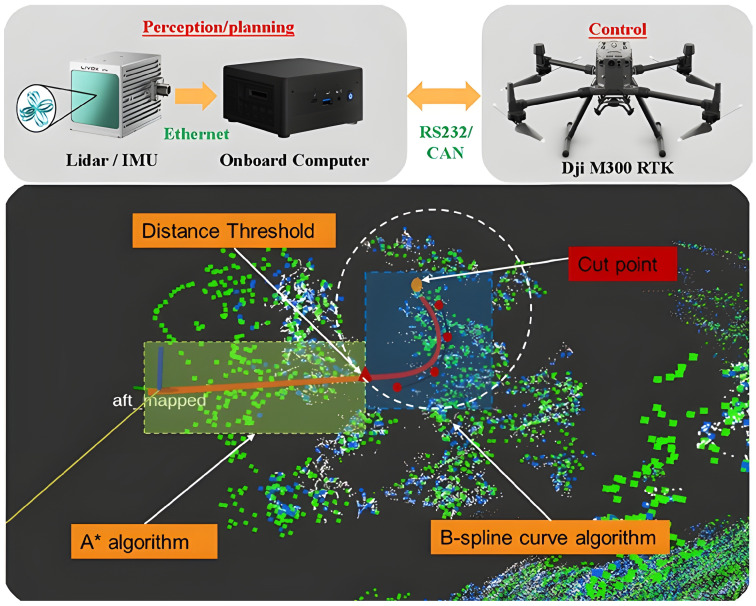
Hardware platform of a fruit-picking UAV and example trajectories generated by a B-spline-based Hybrid A* planner in orchard environments [127].

Conventional planning generally follows a hierarchical framework composed of global planning and local planning [119]. In agricultural operations, global planning is usually based on prior information such as field boundaries, operational areas, no-entry zones, and waypoint tasks, with the objective of generating coverage paths or multi-waypoint visiting routes for applications such as spraying, spreading, and inspection [120,121,122]. Existing reviews have systematically summarized graph-search and sampling-based paradigms, including A*, D*/D* Lite, PRM, RRT/RRT and their variants, while emphasizing the trade-offs among time, energy consumption, and safety constraints [120,123]. From an onboard deployment perspective, graph-search methods such as A* are generally more suitable for structured environments with relatively clear map constraints [124], sampling-based methods such as RRT/RRT* offer stronger adaptability to high-dimensional or kinodynamically constrained planning but often at higher online computational cost [125], whereas DRL-based planners can provide fast inference after training but require substantial training cost and still face challenges in runtime stability and deployment reliability under real agricultural conditions [53]. For 3D agricultural operations, combinatorial planning has also attracted increasing attention, such as combining Hybrid A* with the Traveling Salesman Problem (TSP) to generate efficient 3D trajectories [126]. In more spatially constrained environments such as orchards, planning places greater emphasis on kinematic feasibility and safety-clearance constraints [127]. Accordingly, Hybrid A* is often adopted in practice to combine discrete search with continuous kinematic constraints, thereby producing smoother and executable trajectories [128], and this framework can be extended to 3D trajectories and multi-target tasks [129]. For example, the orchard fruit-picking UAV proposed by Li et al. [127] uses LiDAR to construct a dual static-dynamic map and combines it with an improved B-spline-based Hybrid A* planner to safely reach target clusters while satisfying operational motion constraints, as shown in Figure 7. Local planning, by contrast, typically updates trajectories in real time within a limited sensing range and enforces collision constraints, forming a closed-loop replanning process with global planning. For instance, combining RRT* with the Dynamic Window Approach (DWA) can improve real-time correction capability and energy efficiency in dynamic environments [130]. Such a framework that couples global sampling with local real-time adjustment is directly relevant to agricultural scenarios where predefined operational routes coexist with temporary dynamic obstacles.

Deep learning-based path planning places greater emphasis on end-to-end or near-end-to-end decision-making from perception to control by directly mapping image, depth, or state inputs to velocity commands or action sequences [131,132]. Typical technical routes include deep reinforcement learning, imitation learning, and hybrid architectures that combine learning-based local planners with conventional constraints [133]. For agricultural applications, the key advantage is that under conditions such as visual degradation, complex terrain, or the frequent appearance of targets and obstacles, these methods can generate executable actions within short decision cycles, thereby improving the responsiveness of online planning and control [134]. For example, Wang et al. [134] proposed Land-Automatic Curriculum Learning (Land-ACL), a deep reinforcement learning framework that improves the stability of high-speed autonomous navigation under partially observable conditions, providing a transferable learning-based navigation paradigm for applications such as agricultural irrigation. Overall, learning-based planning is better suited to serve as the local decision or reaction layer, and can be integrated with task-level constraints and safety rules to form a hybrid planning architecture for agricultural UAVs [132].

From an engineering perspective, lightweight path planning and edge-side navigation inference are also critical for agricultural UAV deployment [135]. Because onboard computing power and power supply are both limited, planning modules must generate feasible actions within short decision cycles while maintaining low computational overhead [122,136]. In practice, this makes lightweight local planning, simplified environment representation, and computationally efficient collision checking particularly important for near-ground agricultural navigation. Similarly, when learning-based navigation is deployed onboard, edge-side inference must emphasize low latency, stable runtime, and robustness under varying field conditions, rather than relying solely on computationally intensive models [137]. As a result, future agricultural UAV navigation systems will likely depend on lightweight hybrid architectures that balance planning quality, inference speed, and deployment efficiency. This also reflects a fundamental engineering trade-off in agricultural UAV navigation: higher mapping fidelity, planning complexity, and perception accuracy often improve task performance, but may also increase onboard energy consumption and reduce effective flight endurance under strict payload and battery constraints.

**Figure 8:**
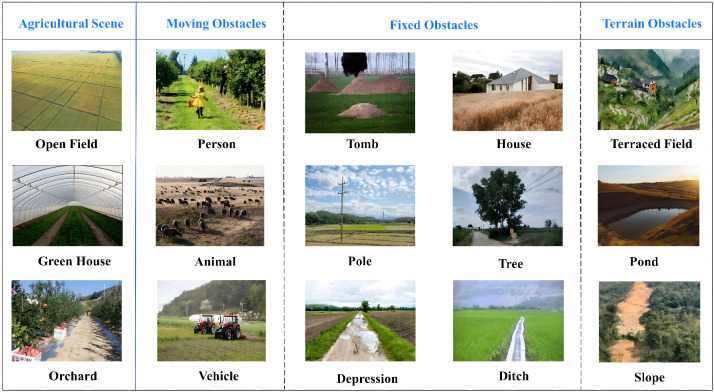
Typical obstacles in agricultural environments [138].

It should be emphasized that obstacle avoidance in this section refers to real-time decision-making at the planning and control levels, rather than the geometric collision representations discussed in the previous mapping section. In agricultural environments, dynamic risks mainly arise from humans, agricultural machinery, vehicles, and animals [138] (Figure 8). A typical pipeline first performs dynamic-object recognition and localization through object detection or semantic segmentation, then transforms the detected objects into safety distances, no-entry regions, or cost terms, and finally generates short-horizon velocity and heading commands through a local planner or policy network to achieve real-time avoidance [139]. In complex agricultural environments, real-time detection frameworks such as YOLO are widely used for dynamic obstacle recognition because of their speed advantages [140,141,142], and are often combined with multi-sensor fusion to improve reliability [138,143]. For example, Zhou et al. [144] proposed an improved YOLOv8-based obstacle detection model that significantly improves detection performance for typical field obstacles while satisfying real-time operational requirements. Studies on orchard fruit-picking UAVs have also demonstrated an engineering pathway based on tight coupling of perception, mapping, and planning, in which LiDAR is used to construct a dual static-dynamic map and planning algorithms are then applied to achieve obstacle-avoidance motion [127].

Visual navigation emphasizes the use of visual cues for localization, trajectory following, and target reaching, and shows particular potential in orchard canopies, greenhouse corridors, and understory environments where GNSS is unreliable [145,146,147]. Building on this, vision-language navigation (VLN) further introduces language instructions by mapping task descriptions to visual understanding and action sequences, thereby providing a more natural human–machine interaction interface for agricultural task scheduling [148]. Recently, SkyVLN integrated VLN with nonlinear model predictive control (NMPC), using large models to interpret instructions and coordinate with the control module to generate feasible trajectories [149,150,151]. In addition, Bhattacharyya et al. [152] proposed the VisNAV framework for open-field scenarios and compared it with GNSS-RTK, VIO, LiDAR, and UWB-based solutions, reporting a localization error of 12–14 cm, which demonstrates the feasibility of visual navigation in agricultural environments.

In large-scale agricultural operations, path planning is increasingly extending from single-UAV navigation to multi-UAV collaborative navigation [153,154]. For representative tasks such as large-area spraying, cooperative inspection, and distributed mapping, multi-UAV systems can substantially improve coverage efficiency, shorten operation time, and alleviate the endurance limitations of individual platforms [155]. From a technical perspective, multi-UAV collaborative navigation typically involves several tightly coupled components, including task allocation, cooperative coverage path planning, formation or spacing maintenance, inter-UAV collision avoidance, communication-aware coordination, and distributed replanning under dynamic field conditions [156]. Compared with single-UAV planning, the main challenge is that navigation is no longer determined solely by environment constraints and vehicle kinematics, but also by coordination consistency across multiple platforms. In agricultural environments, where fields may be spatially large, operational regions may be fragmented, and communication quality may vary across space, these coordination requirements become particularly important. Therefore, multi-UAV collaborative navigation can be regarded as a system-level extension of agricultural UAV planning, in which coverage optimization, safety constraints, and real-time coordination must be jointly considered to support scalable field deployment.

## 3. Applications of UAV Navigation in Agriculture

### 3.1. Aerial Mapping and Field Monitoring

In open-field environments, the integration of UAV navigation and remote sensing is mainly reflected in three task categories: mapping/georeferencing, phenotyping, and monitoring. Compared with close-range scenarios such as orchards, open fields are characterized by regular plot structures and relatively sparse obstacles. Therefore, the main system challenges are typically not narrow-space obstacle avoidance, but coverage completeness, flight repeatability, and cross-temporal georeferencing consistency. In this context, navigation quality is closely tied to high-precision localization and coverage-oriented path planning.

**Figure 9:**
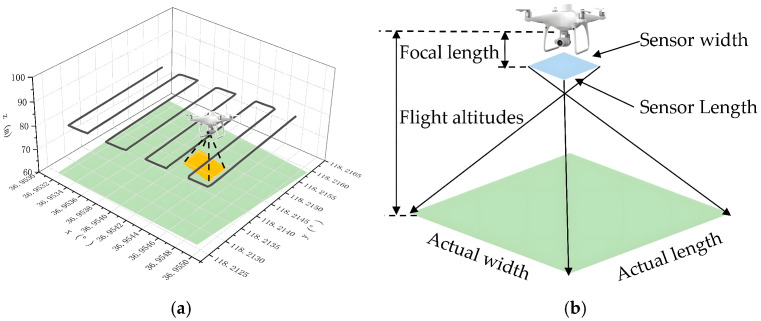
Coverage-oriented flight paths and sensor–ground mapping geometry for open-field aerial surveys [159]: (**a**) representative UAV flight paths over a target field and (**b**) relationship between sensor parameters and ground sampling distance.

For open-field mapping, existing studies mainly focus on the localization and delineation of field plots and experimental subplots for breeding and high-throughput analysis. Han et al. [157] used an A-UNet-based framework for plot extraction from UAV RGB imagery and demonstrated stable performance across different resolutions and plot scales, while earlier work by Luna et al. [158] provided practical evidence for UAV-based mapping in large-scale fields. In such regular farmland settings, mapping quality depends strongly on complete coverage and reduced missed scans, making coverage path planning a natural and effective paradigm. As illustrated in Figure 9, the study by Xue et al. [159] further highlights the coupling among flight-path design, sensor-to-ground imaging geometry, and mapping quality. Accordingly, RTK-supported repeatable flight and high-precision trajectory tracking are particularly suitable for maintaining stable georeferencing of field boundaries, plots, and target objects, thereby establishing a consistent spatial basis for subsequent phenotyping and monitoring tasks.

Beyond spatial mapping, another major open-field application is high-throughput phenotyping, which can be broadly divided into two paradigms: structural or count-based phenotyping, and continuous trait inversion using multi-source remote sensing with machine learning or regression. For structural and count-based phenotyping, Valente et al. [160] combined conventional image processing with CNN-based transfer learning for field plant identification and counting. More recent studies increasingly rely on deep learning for organ-level detection and segmentation. For example, Jia et al. [161] developed a multi-temporal maize tassel detection framework, Peng et al. [162] used Mask R-CNN to extract wheat spike traits and support yield estimation, and Lu et al. [163] proposed a pipeline integrating segmentation, detection, classification, and trait estimation for rice panicle phenotyping. For continuous trait inversion, Zhu et al. [164] systematically compared hyperspectral, thermal, RGB, and LiDAR data for maize trait estimation, showing that different sensing modalities are better suited to different trait types and that multi-source fusion can improve performance under some conditions.

From a navigation perspective, high-throughput phenotyping differs from general mapping in its stronger requirement for repeated revisits to the same trial areas and consistent sampling across growth stages. This places higher demands on flight repeatability, stable low-altitude motion, and local fine alignment. Accordingly, such tasks are better supported by RTK-GNSS for global revisit referencing, combined with vision-based row detection or semantic segmentation and IMU integration for local correction under low-altitude conditions [165]. This hybrid navigation strategy can reduce the effects of canopy appearance changes and environmental disturbances on data consistency, thereby improving both trait comparability and georeferencing stability.

**Figure 10:**
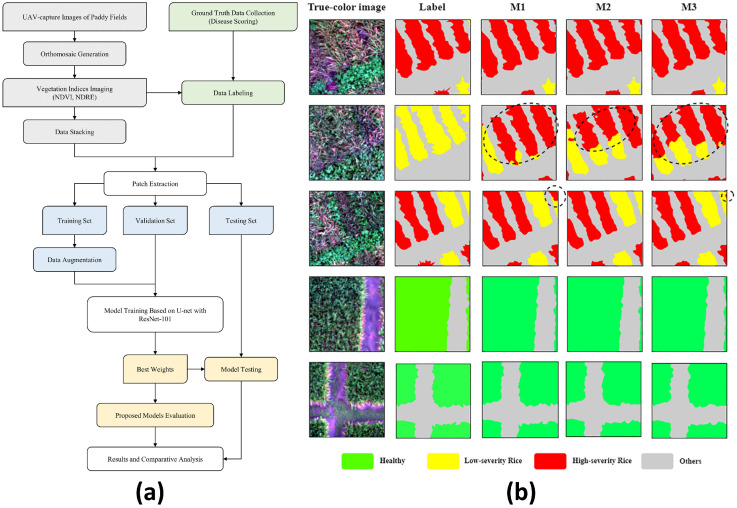
UAV multispectral workflow for rice bacterial leaf blight (BLB) monitoring: (**a**) Workflow of BLB detection in rice field based on UAV multispectral imaging; (**b**) segmentation results for BLB disease in rice field based on UAV image [170].

Open-field monitoring mainly targets the spatiotemporal identification of abiotic stresses, such as water stress, drought, and salinity, as well as biotic stresses such as pests and diseases [166,167]. A common technical paradigm is multispectral or hyperspectral imaging, optionally combined with thermal infrared sensing, followed by feature extraction or deep learning-based analysis and spatial mapping. Representative studies include water-stress estimation from multispectral and thermal imagery [168], salinity estimation using multi-source remote sensing and machine learning [169], and disease monitoring based on UAV multispectral imaging and deep segmentation models [170]. As shown in Figure 10, Logavitool et al. [170] demonstrated a UAV multispectral workflow for rice bacterial leaf blight monitoring, highlighting the role of image-based disease segmentation in field-scale stress assessment.

At the navigation level, open-field monitoring is not only a sensing problem but also a task-driven revisiting problem. Because early pest and disease symptoms are often patchy, sparse, and spatially heterogeneous, these tasks are better suited to a two-stage navigation strategy: RTK-supported high-altitude scanning is first used to obtain field-scale risk distributions, after which adaptive replanning is triggered to revisit hotspot regions at lower altitude for detailed verification [171]. This combination of coverage planning and task-driven local revisiting better matches the spatial heterogeneity of field stresses and the need for high-resolution confirmation.

### 3.2. Precision Spraying and Variable-Rate Application

Precision spraying and variable-rate application (VRA) represent a typical transition of agricultural UAVs from remote sensing platforms to operational platforms for agricultural production. Unlike monitoring tasks, spraying is highly position-sensitive: trajectory deviation, altitude and speed fluctuations, and payload variation directly affect droplet coverage, deposition uniformity, and drift risk. As a result, variable-rate spraying systems usually exhibit a closed-loop coupled structure of prescription information or real-time perception, spray-rate regulation, and navigation with trajectory maintenance. In this framework, navigation provides the spatiotemporal basis for prescription execution and spraying consistency, whereas spray-rate control determines whether dosage can reliably vary according to spatial and canopy demands.

**Figure 11:**
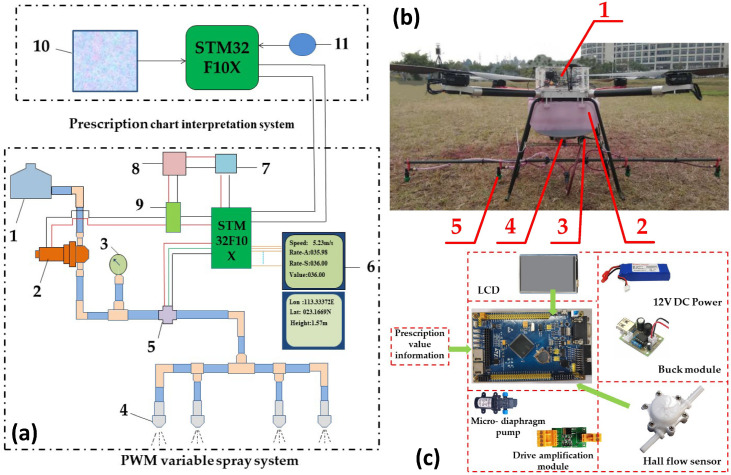
Variable-rate spraying system driven by prescription-map interpretation: (**a**) schematic of prescription interpretation and PWM-based flow control. Note: 1. Medicine box; 2. micro-diaphragm pump; 3. digital pressure gague; 4. pressure nozzle; 5. hall flow sensor; 6. liquid crystal display (LCD); 7. buck module; 8. 12 V direct-current power; 9. drive amplification module; 10. prescription figure; 11. GPS. (**b**) physical implementation of the UAV spraying system. Note: 1. Prescription map interpretation system and spray controller; 2. medicine case; 3. Hall flow sensor; 4. miniature diaphragm pump; 5. pressure nozzle. (**c**) hardware schematic of the spray controller [172].

In open-field scenarios, where field geometry is relatively regular and obstacles are limited, VRA usually takes the form of strip-based full coverage. Accordingly, the main bottlenecks are not complex obstacle avoidance, but coverage completeness, trajectory stability, and closed-loop dosage accuracy. As shown in Figure 11, Wen et al. [172] developed a prescription-driven UAV variable spraying system based on PWM–PID flow regulation, illustrating a practical closed-loop workflow linking map interpretation, flow control, and navigation. As VRA evolves from prescription-zone-based treatment to growth- or state-responsive spraying, real-time perception is increasingly incorporated into the control loop. For example, Liu et al. [173] proposed a LiDAR-assisted variable-rate spraying system that dynamically adjusts operational strategy during flight, further highlighting the dependence of variable spraying on online sensing and navigation consistency.

Spraying operations are also subject to time-varying payloads, model uncertainty, and external wind disturbances, making robust flight control essential for maintaining dosage consistency. In addition, fault-tolerant control has also become increasingly important for plant protection UAVs under actuator failure and operation-induced uncertainties such as time-varying mass and disturbance amplification during spraying [174]. Under such disturbance conditions, Ijaz et al. [175] proposed a hybrid robust control strategy, while Zhang et al. [176] introduced FOSM-ADRC to improve disturbance rejection, suppress chattering, and enhance control response. From a navigation perspective, because open-field spraying emphasizes regular coverage and minimal missed application, coverage path planning supported by RTK-GNSS and IMU provides an appropriate spatial reference for prescription execution and helps reduce the impact of trajectory errors on spraying consistency [173].

As agricultural spraying missions continue to expand in operational scale, multi-UAV collaborative spraying is becoming an important development direction [156,177]. In large fields, collaborative spraying systems can improve operational efficiency through region partitioning, parallel route execution, and cooperative task scheduling, while also reducing the constraints imposed by the limited endurance and payload of a single UAV [155,156]. However, such systems place higher demands on navigation and coordination, since flight trajectories must remain not only individually stable but also mutually consistent across platforms. In this context, multi-UAV collaborative spraying depends on the joint support of cooperative coverage planning, inter-UAV conflict avoidance, timing synchronization, and communication–robust task execution. From a systems perspective, this means that future spraying navigation frameworks should evolve from single-platform trajectory maintenance toward multi-platform coordinated navigation that integrates planning, control, and execution consistency.

**Figure 12:**
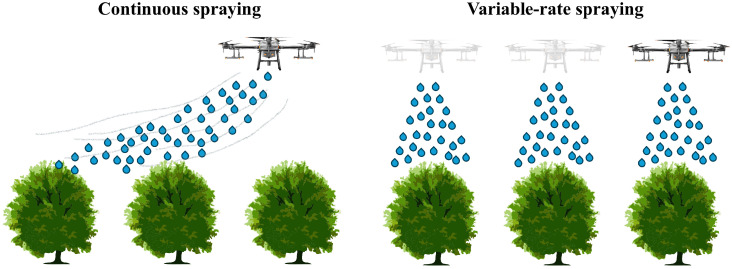
Comparison between continuous-rate spraying and variable-rate spraying in orchard environments, illustrating the motivation for canopy-aware dose modulation [178].

Unlike open fields, orchard VRA must address highly heterogeneous three-dimensional canopy structures. In this context, the objective of variable spraying is not only zonal pesticide allocation, but also dose adaptation to canopy geometry and density. Figure 12 illustrates this shift by comparing continuous-rate and variable-rate spraying in orchard environments. Chen et al. [178] proposed a canopy-volume-based UAV spraying method using LiDAR-derived 3D point clouds to generate differentiated prescription maps, while Zhao et al. [179] combined GNSS positioning with acoustic estimation of canopy leaf area density to enable real-time spray adjustment. Both studies showed that canopy-aware variable spraying can improve within-canopy deposition quality while reducing ground loss and runoff [178,179].

From a navigation perspective, orchard spraying imposes stronger constraints than open-field operation, requiring stable row-following flight, safe canopy-proximal distances, and repeatable trajectories to ensure consistency in 3D canopy perception and prescription execution. Therefore, compared with open-field VRA, which can often rely primarily on GNSS, orchard VRA is better supported by navigation paradigms that integrate GNSS with 3D perception technologies such as LiDAR, thereby mitigating localization degradation caused by canopy occlusion and narrow corridors while enabling closed-loop canopy-aware spraying [178]. More broadly, this indicates that orchard variable spraying depends on tighter coupling among perception, localization, and spray-rate regulation, rather than simply overlaying prescription maps onto a spraying controller.

### 3.3. Orchard Navigation and Close-Range Operations

**Figure 13:**
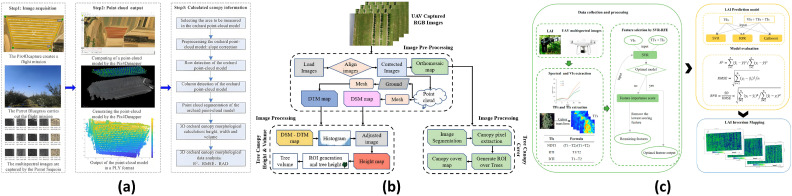
Applications of UAV in orchard environments. (**a**) Operating principle of the UAV canopy photogrammetry system for modern standard orchards [180]; (**b**) Sample workflow for tree canopy characteristics measurements through image analysis [181]; (**c**) Flowchart of UAV multispectral images data analysis and processing (with spectral and texture information) [182].

Orchards and perennial cash-crop planting areas are characterized by pronounced three-dimensional canopy structures, narrow inter-row corridors, and severe occlusion. In such environments, UAVs are used not only for remote sensing observation, but increasingly for canopy-proximal sampling and close-range operational support. Accordingly, navigation is no longer limited to reaching or covering a target area, but instead requires repeatable flight paths, stable relocalization, close-range obstacle avoidance, and consistent attitude control. Because orchard phenotyping and monitoring often depend on highly overlapping imagery and stable flight altitude for cross-temporal alignment, while canopy occlusion and repetitive textures degrade both GNSS and pure vision-based localization, orchard UAV operation places higher robustness demands on the localization–mapping–planning chain. Figure 13 summarizes representative UAV applications in orchard environments and illustrates how these perception and mapping tasks impose scenario-specific navigation requirements.

In orchard environments, UAV mapping commonly targets canopies and fruits for spatial localization and georeferencing to support precision management [180,183]. As illustrated in Figure 13a, canopy-scale mapping methods such as that of Sun et al. [180] rely on photogrammetric point-cloud reconstruction and row-structured representation, which in turn require stable headings and repeatable observation geometry along tree rows. This creates a natural coupling between orchard mapping and row-constrained navigation. To improve stable flight and repeatable sampling under GNSS degradation, recent studies have mainly followed two directions: vision-based control or learning-based navigation [184], and fusion-based localization [185]. These methods are better aligned with the orchard-specific challenge that absolute localization may degrade under occlusion, while the tasks still require repeatable trajectories and stable relocalization.

Another important direction is fruit-level spatial localization, where the objective extends from fruit detection and counting to 3D spatialized yield assessment [126,183,186]. Existing studies have combined video-based detection, hyperspectral sensing, LiDAR mapping, and 3D planning to support fruit localization, repeated-count suppression, and spatial yield estimation [126,183,186]. The navigation implication is that, once orchard tasks move from coverage-oriented observation to near-target multi-view inspection and multi-point access, localization and planning must explicitly support viewpoint repeatability, safe near-obstacle motion, and spatially aware trajectory generation. Such applications are therefore better supported by fusion-based localization and 3D planning frameworks, particularly those constrained by LiDAR geometry or hybrid SLAM/GNSS localization.

Orchard phenotyping tasks often focus on canopy structure, flower and fruit density, and fruit morphology, all of which impose strong constraints on image overlap, viewpoint consistency, and altitude stability. Representative studies include canopy-structure estimation from UAV RGB imagery and photogrammetric reconstruction [181,187], flower-density mapping based on RGB imagery and point clouds [188], and finer-scale 3D fruit phenotyping through panoramic mapping and shape reconstruction [189]. As shown in Figure 13b, these tasks require repeatable cruising along tree rows with stable canopy-relative altitude and consistent viewpoints to ensure cross-temporal comparability and accurate spatial registration. Accordingly, orchard phenotyping is better matched to navigation paradigms constrained by tree-row centerlines, such as LiDAR–IMU fusion methods that support inter-row navigation, headland turning, and repeatable data acquisition under canopy occlusion [190].

Orchard monitoring tasks likewise depend on multi-temporal repeated sampling and stable relocalization to maintain temporal alignment and spatial consistency. Figure 13c shows a representative multispectral analysis workflow for orchard monitoring [182]. Existing studies have used UAV multispectral or high-resolution imagery for leaf area index estimation [182], water-status and transpiration mapping [191], disease detection [192], and yield estimation [193]. Because these tasks require repeated acquisition along similar flight paths across time, they are better supported by tree-row centerline-constrained navigation [190], LiDAR–IMU fusion [194], or SLAM/GNSS fusion and 3D SLAM frameworks [185,195], which improve trajectory stability, relocalization, and cross-flight map consistency under weakened or occluded GNSS conditions. More broadly, orchard monitoring highlights that, in close-range agricultural environments, navigation quality directly affects not only flight safety but also the spatial consistency and interpretability of long-term monitoring outputs.

## 4. Datasets, Simulation, and Evaluation Protocols

### 4.1. Data Fusion and Datasets

The core objective of multimodal data fusion in agricultural UAV navigation is to exploit sensor complementarity to improve localization, mapping, obstacle avoidance, and navigation robustness. In this context, fusion should be understood primarily as a navigation-oriented mechanism for enhancing state estimation, environmental perception, and real-time decision-making under occlusion, illumination variation, and GNSS degradation. According to the stage of information flow, fusion strategies are commonly categorized as early fusion, intermediate fusion, and late fusion [143], corresponding to input-level, feature-level, and decision-level fusion, respectively. Early fusion directly integrates multimodal inputs to strengthen cross-modal interaction. Zhao et al. [196] proposed LIF-Seg, a coarse-to-fine camera–LiDAR early fusion network for LiDAR image segmentation with offset correction for spatiotemporal misalignment. Although not developed specifically for UAVs, this paradigm is relevant to low-altitude agricultural UAV navigation, where RGB, LiDAR, and semantic segmentation can support obstacle recognition, risk understanding, and traversable-area segmentation. Intermediate fusion emphasizes feature alignment and joint optimization within a unified state-estimation framework, making it naturally compatible with tightly coupled odometry and SLAM. Zheng et al. [197] proposed FAST-LIVO2, which integrates IMU, LiDAR, and visual measurements through an efficient error-state iterated Kalman filter (ESIKF) for accurate and robust state estimation. Such approaches are particularly suitable for agricultural UAV navigation because they support continuous 6-DoF pose estimation and improved robustness when GNSS becomes unreliable. Late fusion combines independently estimated modality-specific outputs through weighting or consistency constraints, offering modularity and deployment flexibility. Ali et al. [198] proposed a modular late-fusion framework using camera, LiDAR, and radar data for target classification. Although developed in a UGV context, this paradigm is also applicable to embedded agricultural UAV systems by combining the all-weather robustness of radar with the semantic and geometric capabilities of vision and LiDAR for low-altitude risk recognition and obstacle-avoidance triggering. Overall, the significance of these three fusion paradigms lies not in fusion itself, but in their shared ability to improve navigation reliability in heterogeneous agricultural environments.

**Figure 14:**
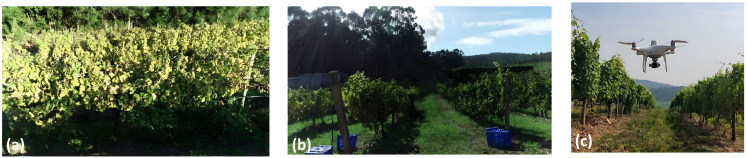
GrapeSLAM dataset [199]: (**a**) side-view video collection; (**b**) front-view video collection; (**c**) UAV position and pose trajectories during flights.

**Figure 15:**
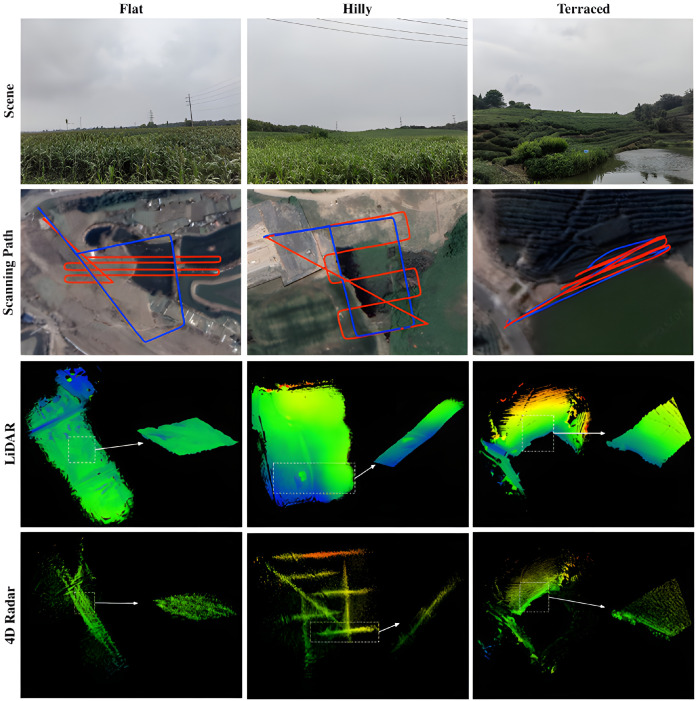
Visualization of the three representative farmland scenarios and their corresponding sensor data [90].

At the data level, the main bottleneck is no longer data scarcity, but the lack of high-quality, standardized, and reproducible datasets for navigation and sensor fusion. For agricultural UAV navigation, such datasets must support rigorous evaluation of localization accuracy, mapping consistency, and robustness across representative field conditions. Dataset construction should therefore provide: (1) strict time synchronization among visual, LiDAR, IMU, and GNSS data; (2) complete calibration, including intrinsic, extrinsic, and temporal parameters; (3) high-quality ground truth, such as RTK/INS, total station measurements, or high-definition maps and trajectories; (4) task-relevant metadata, including flight altitude and speed, crop type, row orientation, occlusion level, illumination, wind conditions, and radiometric settings; and (5) coverage of scenarios with different difficulty levels, such as plains, hills, and terraced fields, as well as weak-texture, repetitive-structure, and strong illumination-variation conditions. GrapeSLAM [199] provides UAV monocular visual data together with RTK/IMU trajectories for vineyard environments (Figure 14), making it suitable for visual SLAM, SfM, and orchard-navigation research. AgriLiRa4D [90], by contrast, contains 33 sequences of LiDAR, 4D radar, and IMU data across plains, hills, and terraced fields (Figure 15), providing a more systematic benchmark for multimodal SLAM and localization in agricultural UAV applications. Overall, these datasets show that future benchmarks should be designed not merely for multimodal sensing, but for navigation-oriented evaluation under representative agricultural disturbances and scene variability. However, current agricultural UAV navigation datasets still exhibit several important limitations. Most available datasets cover only a limited number of crop types, sensing configurations, or operating conditions, and many lack unified calibration standards, sufficiently accurate ground truth, explicit task annotations, or standardized evaluation splits. In addition, cross-dataset comparability remains weak because scenario definitions, sensor combinations, and reporting protocols are often inconsistent. As a result, although recent datasets have improved data availability, they still fall short of supporting fully standardized and reproducible benchmark evaluation for agricultural UAV navigation.

### 4.2. Simulation and Digital Twin

**Figure 16:**
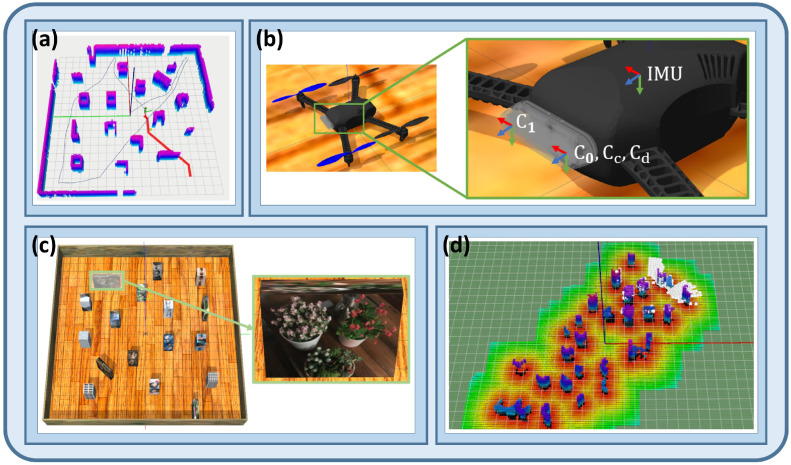
Example of UAV navigation in the Gazebo simulator [206]: (**a**) click-and-fly navigation in an unknown environment (blue: executed trajectory; red: planned global path); (**b**) UAV model and on-board sensors; (**c**) a 20 × 20 m world with obstacles and feature-rich textures; (**d**) visualization of global/local/ESDF maps in Rviz for closed-loop navigation.

Simulation is a fundamental tool for validating safety, expanding data scale, and accelerating iteration in UAV navigation and control research. However, different platforms emphasize different aspects: control-loop and dynamic accuracy, ROS-based closed-loop multi-sensor integration, and high-fidelity perception with large-scale training often require different software ecosystems. In general, MATLAB is more suitable for flight dynamics and control-loop modeling [200,201], Gazebo is better suited to ROS-based closed-loop validation and multi-sensor integration [202], and Isaac Sim is more appropriate for high-fidelity perception simulation, synthetic data generation, and large-scale training [203]. In agricultural UAV applications, MATLAB is commonly used for flight control and autopilot or hardware-in-the-loop (HIL) validation [204,205]; Gazebo is frequently used for closed-loop testing of visual SLAM, autonomous navigation, trajectory tracking, and obstacle-avoidance logic [202,206]; and Isaac Sim is more suitable for high-fidelity validation and training of multirotor control and motion-planning algorithms [207,208]. Figure 16 presents a Gazebo-based workflow for click-and-fly navigation in unknown environments, including sensor configuration and ESDF/map visualization in Rviz [206], illustrating a closed-loop validation pipeline of simulation, mapping, planning, and execution.

Digital twins further extend simulation into an updatable virtual–physical interactive system, in which the environment, platform, and task states are continuously synchronized in virtual space, thereby supporting path planning, risk prediction, online validation, and model transfer. Wu et al. [209] combined digital twins with 3D A* planning to validate UAV navigation and obstacle-avoidance processes in a virtual campus environment. For agriculture, this paradigm is particularly suitable for geometry-sensitive close-range scenarios, such as orchard inter-row flight, greenhouse corridors, sloped or terraced fields, and multi-UAV collaborative field inspection, where it can be used to rapidly assess system-level trade-offs among planning strategy, safety margin, and operational efficiency. At the same time, the key to Sim2Real lies in reducing the gap between simulation and the real world. Recent UAV remote sensing studies have improved transferability through high-fidelity scene reconstruction and diversified rendering [210,211], thereby providing a more practical technical pathway for synthetic data generation, Sim2Real, and Sim2Real2Sim in agricultural navigation. However, even high-fidelity simulation cannot fully reproduce the complexity of real agricultural deployment, including wind-driven canopy motion, illumination variation, GNSS instability, spray interference, terrain irregularity, and communication uncertainty [212,213]. Therefore, simulation results should be interpreted primarily as a controllable pre-validation tool, whereas real-world field deployment remains essential for assessing robustness, transferability, and task-level reliability [214,215]. In this sense, the Sim-to-Real gap in agricultural UAV navigation lies not only in perception realism, but also in the mismatch between controlled virtual validation and long-duration operation under coupled environmental and task disturbances.

### 4.3. Evaluation Metrics

Since existing studies on agricultural UAV navigation are conducted under heterogeneous platforms, sensing configurations, task settings, and validation protocols, direct metric-level comparison across all methods is often difficult. Therefore, the purpose of this section is not to impose a single unified ranking, but to summarize the most commonly used and practically meaningful evaluation metrics in the literature, so as to provide a clearer analytical basis for interpreting reported results and to support future benchmark design and more standardized cross-study comparison. As summarized in Table 3, the evaluation of agricultural UAV navigation can be organized into six closely related categories: localization and mapping, trajectory tracking, control performance, mission efficiency, safety and robustness, and agronomic task quality. For localization and mapping, the most widely used metrics are absolute trajectory error (ATE) and relative pose error (RPE), which characterize global trajectory deviation and local drift, respectively, and are commonly used in SLAM, VIO, LIO, and fusion-based localization evaluation [216]. For trajectory tracking, representative metrics include RMSE/MAE and cross-track error, which reflect point-wise tracking deviation and lateral line-following quality in tasks such as waypoint following, spraying route tracking, and crop-row navigation [217]. For control performance, settling time and overshoot are commonly used to evaluate response speed and dynamic stability under disturbances, especially for PID-, sliding-mode-, ADRC-, and MPC-based controllers under wind disturbance or time-varying payload conditions [135]. At the mission level, path length and flight time provide compact measures of planning efficiency and operational cost, while success rate, collision rate, and minimum clearance are important for assessing navigation safety and robustness in cluttered or uncertain environments [218]. In addition, because agricultural UAVs are ultimately designed for field operations rather than navigation alone, agronomic task quality must also be considered. Typical indicators include coverage rate, miss rate, overlap rate, spray deposition density, deposition uniformity measured by coefficient of variation (CV), and drift loss, which help translate navigation and control performance into differences in actual operational quality.

## 5. Discussion

### 5.1. Challenges

The high heterogeneity of agricultural environments makes it difficult to develop universally transferable UAV navigation solutions. Compared with general low-altitude scenarios, agricultural environments differ markedly in spatial structure, terrain variation, crop morphology, occlusion conditions, and task objectives [1,219]. Open fields emphasize large-area coverage and trajectory repeatability, orchards require canopy-proximal perception and stable flight in narrow corridors, and terraced or mountainous farmlands impose stricter demands on terrain adaptation and flight safety. This strong coupling among scenario, task, and navigation requirements means that existing methods often perform well only under specific conditions and are difficult to transfer reliably across crop types, seasons, and operational modes [124]. Therefore, the key challenge in agricultural UAV navigation is not the isolated performance of individual algorithms, but the lack of a systematic methodology that links environmental characteristics, task requirements, and navigation architecture selection. This also limits the scalability and real-world applicability of current navigation systems, since methods validated in one agricultural scenario often do not transfer reliably across different crops, terrains, and operational conditions.

Perception degradation and unstable localization remain central bottlenecks to robust autonomous navigation in agriculture. Agricultural environments commonly involve weak or repetitive textures, dynamic vegetation, severe illumination variation, GNSS occlusion, and disturbances from dust and airflow [220,221,222]. These factors jointly degrade visual localization, GNSS/RTK positioning, and low-altitude state estimation. More importantly, localization errors propagate beyond the state-estimation layer into mapping, replanning, trajectory control, and task execution, ultimately affecting spraying uniformity, phenotyping consistency, and revisit accuracy [223]. Although multi-sensor fusion improves robustness, its field deployment remains constrained by time synchronization, extrinsic calibration, payload, energy, and computational limitations. As a result, agricultural UAVs still lack localization solutions capable of reliable long-duration operation in complex environments.

Another major challenge is the disconnect between environmental representation, path planning, and agricultural task objectives in current navigation pipelines. Most existing studies still design navigation systems primarily around geometric traversability, collision avoidance, or path executability [224], whereas agricultural operations are more directly evaluated by outcome-oriented indicators such as spraying deposition consistency, stable canopy-relative distance, cross-temporal route reproducibility, and efficient revisiting of pest or disease hotspots [154,225]. Consequently, a geometrically feasible path is not necessarily optimal for agricultural operation. For example, a trajectory that satisfies obstacle-avoidance constraints may still be unsuitable for canopy-proximal variable spraying, while a globally complete coverage route may not support adaptive revisiting of hotspot regions. Overall, current agricultural UAV navigation remains largely geometry-driven, while task semantics such as crop rows, canopy boundaries, risk regions, and no-spray zones are still insufficiently incorporated. A truly closed-loop navigation framework oriented toward agricultural task quality has not yet been established.

Time-varying payloads, wind disturbances, and near-ground operating conditions further increase the robustness requirements of agricultural UAV control systems. During spraying, canopy-proximal inspection, and terrain-following tasks, liquid consumption, liquid sloshing, crosswind disturbances, and low-altitude aerodynamic effects all increase dynamic uncertainty, making conventional fixed-parameter control strategies prone to performance degradation [226,227]. Unlike general UAV missions, control in agricultural scenarios is not only concerned with flight stability, but also directly affects canopy-relative height maintenance, sensing geometry, and spraying-quality consistency [228]. Therefore, the challenge of agricultural UAV control lies in maintaining both state stability and task-output stability, which requires control objectives to extend beyond attitude and position regulation toward robust closed-loop control for operational quality.

Finally, the lack of standardized datasets, unified evaluation protocols, and task-oriented benchmarks limits the comparability and reproducibility of agricultural UAV navigation research. Although several navigation and multi-sensor datasets have emerged for agricultural scenarios, they still suffer from incomplete modality coverage, limited scenario diversity, insufficient ground truth, and scarce task annotations. More importantly, many studies rely on private datasets, heterogeneous platforms, and inconsistent evaluation metrics, making fair comparison across methods difficult [229]. Existing evaluations still focus mainly on localization error, path length, and success rate, while giving insufficient attention to agriculture-specific indicators such as spraying deposition uniformity, revisit consistency, and safety margins in canopy-proximal operations. As a result, the relationship between navigation performance and agricultural task outcomes remains unclear, slowing the transition of agricultural UAV navigation from algorithmic validation to engineering maturity.

### 5.2. Future Perspectives

Future development of agricultural UAV navigation should shift from single-scenario optimization to systematic architecture design that explicitly accounts for scenario differences. Given the substantial differences among open fields, orchards, and terraced farmlands in spatial structure, occlusion characteristics, and operational objectives, future research should no longer pursue a single universal solution. Instead, it should establish a navigation technology selection framework oriented toward environmental structure and task attributes, in which sensor configuration, localization strategy, map representation, planning method, and control architecture are co-designed. The key is not to identify one optimal algorithm for all scenarios, but to develop a modular navigation system that can be adaptively configured, rapidly deployed, and stably transferred according to the characteristics of agricultural environments.

Future research must also further develop multi-source robust perception and fault-tolerant localization mechanisms for complex agricultural environments. In addition, future agricultural UAV navigation should move toward lightweight, energy-aware, and edge-deployable architectures to support robust localization, planning, and inference under realistic onboard resource constraints [91,230]. At the methodological level, deeper integration of GNSS, vision, LiDAR, IMU, radar, and environmental sensing information is needed to improve the stability of state estimation under weak GNSS, dynamic occlusion, and low-texture conditions [231,232,233]. However, a major gap in current multi-sensor fusion research is that many methods are still developed and evaluated under relatively controlled conditions, while agricultural environments often involve asynchronous sensing, temporary sensor degradation, calibration drift, canopy motion induced by wind, dust or spray interference, and intermittent GNSS blockage. Future fusion frameworks should therefore move beyond accuracy-oriented integration toward uncertainty-aware, fault-aware, and recovery-oriented architectures that can support sensor reweighting, adaptive modality switching, and degradation under dynamic field conditions. At the system level, greater attention should be paid to engineering issues such as time synchronization, extrinsic calibration, fault detection, and switching under degraded operating conditions [234,235]. In particular, the localization system of agricultural UAVs should not focus solely on improving average accuracy, but should emphasize recoverability and robustness during long-duration operations and under complex disturbances. In other words, the goal should evolve from high-precision localization to high-reliability localization.

In perception and environmental modeling, future research may increasingly incorporate general-purpose visual foundation models, such as the DINO series [236,237,238,239] and the SAM series [240], to improve representation generalization and task transferability in agricultural scenarios. Compared with conventional data-driven approaches that depend heavily on task-specific annotations, these models offer potential advantages in weakly supervised representation learning, open-scene segmentation, cross-domain feature extraction, and few-shot adaptation. As a result, they may provide more transferable visual priors for crop-row extraction, canopy-boundary perception, obstacle recognition, traversable-area segmentation, and hotspot localization of disease symptoms. When combined with 3D reconstruction, dense depth estimation, or multi-view geometric modeling [241], they may further support 3D semantic mapping for structural understanding of agricultural environments, thereby improving the modeling of complex canopies, sloped terrain, and low-altitude traversable space. However, their deployment on agricultural UAV platforms still depends on addressing key challenges related to computational cost, real-time performance, cross-season generalization, and multimodal adaptation. Therefore, these models should be regarded as enhancement modules for high-level environmental understanding and task perception, rather than direct replacements for existing navigation pipelines.

Future navigation systems should also evolve toward task-semantic-driven and closed-loop decision-making. Agricultural UAV navigation should not remain limited to geometric maps and feasible paths, but should explicitly incorporate task semantics such as crop rows, canopy structures, pest and disease hotspots, prescription regions, and safety-risk zones into map representations, cost functions, and replanning logic, thereby forming a closed-loop chain of environmental understanding, task planning, and execution feedback. For typical applications such as spraying, monitoring, and phenotyping [242,243], this means that future systems must advance from simply being able to avoid obstacles and reach targets to being able to make optimal decisions around agricultural objectives, thereby unifying navigation performance with operational quality. Another important direction is the tighter integration of UAV navigation with agricultural decision-support systems. In future workflows, prescription maps, disease-risk maps, phenotyping priorities, and stress-monitoring outputs should not merely serve as offline references, but should directly inform route generation, sensing resolution allocation, revisiting strategy, and mission scheduling. Such integration would enable agricultural UAV navigation to evolve from reactive path execution toward decision-informed and agronomy-aware mission adaptation.

At the control level, agricultural UAVs need to evolve from conventional trajectory-tracking control toward intelligent control frameworks that integrate robustness with task adaptability. ADRC is particularly promising in agricultural scenarios [244,245], because it can estimate and compensate for total disturbances online through an extended state observer under model uncertainty, strong external disturbances, and significant parameter variation [246,247]. This makes it well suited for the complex uncertainties associated with spraying payload changes, wind disturbances, and low-altitude flight [248]. Further integration of ADRC with model predictive control, learning-based control, or multi-sensor state feedback may enable control architectures that jointly satisfy fast response, disturbance rejection, and task-output constraints [249,250]. More broadly, from a cybernetics perspective, agricultural UAV navigation should be understood as a closed-loop adaptive system spanning perception, decision-making, execution, and feedback. Its central challenge lies not only in the local performance of individual controllers, but also in the self-correcting and continuously optimizing capability of the overall system under dynamic conditions.

Future studies should also accelerate the development of standardized data, simulation, and evaluation benchmark systems for agricultural UAV navigation. This requires not only high-quality multimodal public datasets [229], but also unified calibration standards, real trajectory ground truth, representative task definitions, and cross-scenario evaluation protocols. Furthermore, evaluation frameworks should move beyond isolated navigation metrics toward joint assessment of localization accuracy, trajectory stability, task efficiency, safety margins, and agricultural operation quality, so that navigation performance can be interpreted in relation to spraying quality, phenotyping consistency, and disease revisit effectiveness. In particular, future benchmarks should explicitly evaluate robustness under dynamic agricultural conditions, such as wind-driven canopy motion, illumination variation, intermittent GNSS availability, sensor dropout, moving obstacles, and time-varying payloads during spraying. This is essential for narrowing the gap between algorithmic validation under idealized conditions and reliable deployment in real agricultural operations. In addition, reproducible test loops based on high-fidelity simulation, digital twins, and Sim-to-Real technologies [214,215] will provide an important foundation for advancing agricultural UAV navigation from laboratory validation to large-scale deployment.

## 6. Conclusions

This paper has systematically reviewed UAV navigation in agricultural environments from a system perspective, covering key technical components including perception, localization, mapping, planning, control, and data and evaluation frameworks. It has further analyzed the differences in navigation requirements and application patterns across representative scenarios such as open fields, orchards, and terraced farmlands. The review shows that agricultural UAV navigation is no longer a standalone problem of localization or path planning, but rather a comprehensive technical system deeply coupled with operational objectives, environmental structure, and system constraints. Its performance affects not only flight safety and trajectory stability, but also spraying quality, monitoring accuracy, phenotyping consistency, and revisit effectiveness. At present, the field still faces several critical challenges, including strong scenario heterogeneity, significant perception degradation, insufficient integration of task semantics, limited control robustness, and the lack of standardized benchmarks. In the future, agricultural UAV navigation should move beyond local optimization for individual scenarios toward systematic architecture design under the joint constraints of complex environments and agricultural tasks. Through the combined advancement of multi-source robust perception, task-semantic-driven decision-making, intelligent control, and standardized evaluation systems, agricultural UAVs can evolve from being merely flyable and observable to becoming highly reliable autonomous systems that are stable, interpretable, and scalable for real agricultural operations.

## Figures and Tables

**Table 1:** Comparison of localization methods in agricultural UAV navigation.

Category	Representative Methods	Advantages	Limitations	Typical Agricultural Applications
GNSS-RTK	Onboard RTK, VRS, and EKF-based fusion [72,73]	Centimeter-level absolute positioning for trajectory tracking, precision spraying, and direct georeferencing [77]	Sensitive to occlusion and interference; dependent on base stations or network connectivity	Open-field autonomous operation; aerial surveying and mapping
VIO/SLAM	OKVIS, VINS, and ORB-SLAM3 [85,88]	High-frequency, smooth pose estimation; usable in GNSS-denied environments	Degrades under challenging visual conditions; drift accumulates over long distances	Orchard inspection, inter-row navigation, and greenhouse operations
LIO	FAST-LIO [89]	Robust in weak-texture and dynamic vegetation environments	Higher cost, payload, and system complexity	Low-altitude flight beneath orchard canopies and in complex environments
Altitude estimation	Fusion of barometric, GNSS, and ranging measurements [82]	Improves canopy-relative altitude stability	Requires careful fusion tuning; reflective surfaces may affect ranging accuracy	Constant-height spraying and terrain-following flight

**Table 2:** Comparison of mapping representations for agricultural UAV navigation.

Method Category	Representative Methods	Advantages	Limitations	Computational Characteristics	Typical Agricultural Applications
Occupancy/Voxel Maps	OctoMap [102]	Compressed structure for large-scale 3D storage and query	No explicit distance information for trajectory optimization	Moderate cost; memory-efficient, but update/query cost grows with scene scale	Farmland obstacle avoidance; accessibility assessment of roads or irrigation channels
Distance Field Maps	Voxblox [104], FIESTA [106]	Provides distance and gradient information for online trajectory optimization	High computational and memory overhead	Higher online update burden than occupancy maps	Orchard corridor flight; canopy safety-distance constraints
Sparse Visual Maps	ORB-SLAM2 [109]	Efficient for real-time localization	Sparse geometric representation	Lightweight and suitable for onboard deployment, but geometrically limited	Orchard inspection and inter-row navigation
Dense/Semi-dense Maps	KinectFusion [110], LSD-SLAM [112]	Continuous geometry for fine-grained obstacle avoidance	High computational demand	High memory and processing cost; less suitable for long-duration edge deployment	Canopy-proximal operations and near-ground inspection
Semantic Maps	Kimera [113]	Combines geometry and semantics for task-oriented decision-making	Depends on semantic models	Additional inference cost from semantic labeling	Crop-row recognition and risk-area annotation
Dynamic Mapping	DynaSLAM [118]	Removes dynamic objects from maps	High computational complexity	High real-time cost for detection, masking, and reconstruction	Dynamic obstacle avoidance involving machinery, humans, and animals
LiDAR Point Processing	Ground segmentation [114], point cloud filtering [115], farmland point cloud separation [117]	Improves map quality	Sensitive to parameters and scene conditions	Moderate to high preprocessing cost depending on point-cloud density and update rate	Terrace terrain modeling and farmland obstacle extraction

**Table 3:** Evaluation metrics for UAV navigation in agriculture, covering localization/mapping, tracking/control, mission efficiency, safety/robustness, and task-specific agronomic quality.

Metric Category	Common Metrics	What It Measures	Typical Tasks
Localization & Mapping	ATE [216]	Global trajectory deviation from ground truth	SLAM, VIO/LIO, GNSS/INS fusion
	RPE [216]	Local relative pose drift over short intervals	VO/VIO, short-term stability
Trajectory tracking	RMSE/MAE [217]	Point-wise tracking error to a reference trajectory	Waypoint tracking, spraying line following
	Cross-track error [217]	Lateral deviation from a reference line (line-following quality)	Coverage flight, row-following, variable-rate execution
Control performance	Settling time/Overshoot [135]	Dynamic response speed and overshoot under disturbances	PID/SMC/ADRC/MPC, payload/wind disturbances
Mission efficiency	Path length/Flight time [218]	Mission cost and time-to-completion	Planning, autonomous inspection, coverage missions
Safety & robustness	Success rate/Collision rate/Minimum clearance [218]	Completion reliability and safety margins	Autonomous navigation in cluttered/unknown environments
Agronomic task quality	Coverage rate/Miss rate/Overlap rate	Completeness and redundancy of field operations	Mapping, spraying, variable-rate application
	Deposition density/CV/Drift loss	Spray deposition quality and environmental loss	Precision spraying and VRA

Abbreviations: ATE, absolute trajectory error; RPE, relative pose error; RMSE, root mean square error; MAE, mean absolute error; CV, coefficient of variation; VRA, variable-rate application.

## Data Availability

No new data were created or analyzed in this study.

## Appendix A. Review Methodology

This review adopted a structured literature-survey approach to synthesize research on UAV navigation in agricultural environments from a system perspective. Relevant studies were primarily collected from Web of Science, Scopus, IEEE Xplore, and Google Scholar. The search mainly focused on literature published from 2018 to 2026, while earlier seminal studies were retained when they were necessary to describe foundational methods or benchmark frameworks. Representative search terms included combinations of “UAV”, “drone”, “agriculture”, “navigation”, “localization”, “mapping”, “SLAM”, “path planning”, “trajectory tracking”, “obstacle avoidance”, and “spraying”, together with related terms such as “multimodal fusion”, “digital twin”, and “edge deployment”.

The collected literature was screened according to its relevance to agricultural UAV navigation and to the system-level scope of this review (Table A1). Studies were included when they addressed core navigation-related topics, such as sensing, localization, mapping, planning, control, datasets, simulation, or evaluation, in agricultural UAV scenarios or in closely related technical contexts with clear transferability. Studies were excluded when they focused only on general remote sensing analysis without clear navigation relevance, on non-UAV systems without transferable insight, or on papers lacking sufficient technical detail. The final literature set was then organized according to the main structure of this review, including key technologies, representative applications, and supporting datasets, simulation platforms, and evaluation protocols.

**Table A1:** Inclusion and exclusion criteria for literature selection in this review.

Criterion Type	Description
Inclusion	Studies directly related to agricultural UAV navigation or to key navigation-enabling components, including sensing, localization, mapping, SLAM, path planning, obstacle avoidance, trajectory tracking, control, datasets, simulation, and evaluation.
	Studies conducted in representative agricultural scenarios, such as open fields, orchards, and terraced or mountainous farmland, or technically related contexts with clear relevance to agricultural UAV navigation.
	Seminal earlier studies retained when they provide foundational methods or widely used benchmark frameworks.
Exclusion	Studies focused only on crop monitoring or remote sensing interpretation without clear relevance to UAV navigation.
	Studies on non-UAV platforms or non-agricultural systems without sufficiently transferable navigation insight.
	Papers lacking clear technical content, methodological detail, or relevance to the system-level focus of this review.
